# Structure-Based Primer Design Minimizes the Risk of PCR Failure Caused by SARS-CoV-2 Mutations

**DOI:** 10.3389/fcimb.2021.741147

**Published:** 2021-10-25

**Authors:** Hongjie Dong, Shuai Wang, Junmei Zhang, Kundi Zhang, Fengyu Zhang, Hongwei Wang, Shiling Xie, Wei Hu, Lichuan Gu

**Affiliations:** ^1^ State Key Laboratory of Microbial Technology, Shandong University, Qingdao, China; ^2^ Research and Development Department, Shandong Shtars Medical Technology Co., Ltd, Jinan, China

**Keywords:** SARS-CoV-2, quantitative reverse-transcription PCR, variants, false-negative, thermal stability

## Abstract

The coronavirus disease 2019 (COVID-19) has caused and is still causing tremendous damage to the global economy and human health. Qualitative reverse transcription-PCR (RT-qPCR) is the golden standard for COVID-19 test. However, the SARS-CoV-2 variants may not only make vaccine less effective but also evade RT-qPCR test. Here we suggest an innovative primer design strategy for the RT-qPCR test of SARS-CoV-2. The principle is that the primers should be designed based on both the nucleic acid sequence and the structure of the protein encoded. The three nucleotides closest to the 3′ end of the primer should be the codon which encodes the tryptophan in the structure core. Based on this principle, we designed a pair of primers targeting the *nucleocapsid* (*N*) gene. Since tryptophan is encoded by only one codon, any mutation that occurs at this position would change the amino acid residue, resulting in an unstable N protein. This means that this kind of SARS-CoV-2 variant could not survive. In addition, both our data and previous reports all indicate that the mutations occurring at other places in the primers do not significantly affect the RT-qPCR result. Consequently, no SARS-CoV-2 variant can escape detection by the RT-qPCR kit containing the primers designed based on our strategy.

## Introduction

Since its outbreak in December 2019 in Wuhan, China, the coronavirus disease-2019 (COVID-19) soon developed into a global pandemic (Liu R. et al., 2020; Wang et al., 2020a). As of July 14, 2021, more than 187.09 million COVID-19 cases and 4.04 million deaths had been confirmed, giving an overall mortality rate of 2.16%. Severe acute respiratory syndrome coronavirus 2 (SARS-CoV-2) was identified to be the causative agent. Compared to its sister virus SARS-CoV which caused the 2003 SARS epidemic (8,422 cases), SARS-CoV-2 is much more contagious (Liu Y. et al., 2020; Wu et al., 2020). Its R0 value is 2.3, but it could be as high as 5.78 (Bulut and Kato, 2020). Rapid diagnosis and isolation are the first and most effective steps to inhibit the spread of SARS-CoV-2 before the vaccines, and therapeutic drugs are sufficiently effective to control the pandemic (Wang et al., 2020d; Younes et al., 2020). At present, the diagnostic methods for COVID-19 mainly include nucleic acid detection (RT-qPCR), antigen testing, and imaging technology (Jin et al., 2020). Because RT-qPCR has the characteristics of short window period, high sensitivity, and high specificity, it has become the golden standard for COVID-19 test in various institutions around the world (Liu R. et al., 2020; Udugama et al., 2020; Wang et al., 2020b).

It is noteworthy that SARS-CoV-2, as an RNA virus, utilizes RNA-dependent RNA polymerase (RdRp) for the replication and transcription of its genome (Gao et al., 2020). Despite the existence of a “proofreading” mechanism based on nsp14 exonuclease (ExoN) to remove mistakenly incorporated nucleotides, variant strains are still constantly accumulating (Lippi et al., 2020; Wang et al., 2020c). According to the GISAID database, thousands of single-nucleotide variants have been identified across different SARS-CoV-2 strains isolated. Four main types of variants—D614G, Cluster 5, VOC 2020/12/01, and 501Y.V2—have acquired wide attention. The current epidemiological studies have shown that, although these variants do not increase the severity of the disease (assessed by hospitalization time and 28-day mortality) and reinfection, they lead to higher morbidity and hospitalization and more death cases (Abdullahi et al., 2020; Davies et al., 2020; Korber et al., 2020; Tegally et al., 2020; Oude Munnink et al., 2021). It also raises a concern that, as more and more mutations accumulate, some variants may evade the RT-qPCR test, leading to more and more false-negative results (Lippi et al., 2020; Wang et al., 2020b; Rahman et al., 2021). A previous study showed that using degenerate primers in PCR is helpful to detect the variants (Li et al., 2015; Campos and Quesada, 2017; Li et al., 2020), but using degenerate primers brings high cost and low amplification efficiency, which prevents its wide use, especially in cases of detecting positive critical samples (Souvenir et al., 2007; Maher-Sturgess et al., 2008). Therefore, it is an urgent task to find a way to minimize the risk of PCR failure caused by SARS-CoV-2 mutations.

A phylogenetic analysis of the genome sequence of SARS-CoV-2 revealed that *ORF1ab*, *E*, and *N* genes are highly conserved in sarbecoviruses and have been used as target genes by Centers for Disease Control and Prevention (CDCs) in various countries for RT-qPCR test (Guan et al., 2020; Ramirez et al., 2020; Rahman et al., 2021). By far, all these target genes have undergone mutations, with the *N* gene having the greatest number of mutations on the targets of primers and probes which have been widely used around the world to diagnose COVID-19 (Wang et al., 2020d). Here we systematically analyze the effect of the mismatches between the target sequences and primers or probes on PCR test. Consistent with previous reports, the mismatches occurring at the 3′ end of the primer have the greatest impact on the PCR reaction (Bru et al., 2008; Stadhouders et al., 2010). Therefore, placing the 3′ end of the primers at the site where any mutation is lethal to the virus would greatly reduce the risk of PCR failure caused by SARS-CoV-2 mutations. It has been reported that tryptophan, as the largest amino acid, has a special bulky side chain and plays an important role in protein structure stability (Santiveri and Jiménez, 2010; Bielecki et al., 2014). Among the 20 amino acids that make up proteins, only tryptophan and methionine are encoded by a non-degenerate codon. Combining this information, we suggest an innovative structure-based primer design strategy for the RT-qPCR test of SARS-CoV-2. Experiments showed that the primers designed based on this strategy have the same specificity and sensitivity as the primers designed by US and Chinese CDCs. Mutations affecting the RT-qPCR test always lead to the production of an unstable nucleocapsid, which means that these kinds of SARS-CoV-2 variants cannot survive.

## Materials and Methods

### Bacterial Strains, Plasmids, and Culture Conditions

The *N* genes of seven coronaviruses (SARS-CoV, MERS-CoV, HCoV229E, HCoV-OC43, HCoV-NL63, HCoV-HKU1, and SARS-CoV-2) that infect humans were synthesized in Beijing Genomics Institute and then inserted into a pUC57 vector, respectively. The *N* gene of SARS-CoV-2 was cloned into a PET28a vector. All these plasmids were then transformed into *Escherichia coli* DH5α cells for amplification. Plasmids containing the mutant N protein gene were constructed using the improved QuikChange method (Xia and Xun, 2017). The partially overlapping primers were used to generate the PCR products for site-directed mutagenesis (SDM). Then, Fast-Digest *Dpn*I was added into the SDM–PCR reaction mix directly without purification. The mixture was incubated at 37°C for 1–3 h to completely remove the residual template plasmid. Furthermore, 5–10 μl *Dpn*I-treated SDM–PCR product was added into the competent cells for DNA transformation. The positive clones were identified by DNA sequencing. Plasmid DNA was extracted by using Plasmid Mini Kit (Omega) and saved at -20°C for future use. For protein expression, plasmids containing the SARS-CoV-2 *N* gene and mutants were transformed into *E. coli* BL21 (DE3).

### Preparation of the RT-qPCR Template

The plasmids containing the *N* genes of the other six types of coronaviruses and human genome were prepared. These plasmids and *N* gene pseudovirus were added to the throat swab from healthy human and placed into the preservative solution (25 mM Tris–HCl, pH 7.6, 1 mM EDTA, 20 mM guanidine thiocyanate) to simulate inactivating clinically positive samples. Then, 200 μl of preservation solution was taken, and the RNA/DNA was extracted using MiniBEST Viral RNA/DNA Extraction Kit (TaKaRa). The DNA template is diluted with enzyme-free water to 10^7^copies/ml, and the RNA is diluted with enzyme-free water to 10^7^, 10^6^, 10^5^, 10^4^, 10^3^, 700, 500, 400, and 300 copies/ml.

### Effect of Mismatch Between Primers and Template on RT-qPCR Reaction

Three pairs of primers designed by the US CDC were chosen as the research object, with the PUC57-N plasmid as the template. A series of mutations was designed on the primers to create mismatches with the template DNA. The mutations change the first, the second, and the third nucleotide of the 3′ end of the primers and one nucleotide in the middle of the primers, respectively. At each position the nucleotide was mutated into another three mismatched nucleotides (for example, A was mutated to T, C, and G). Then, the Ct values for the RT-qPCR reactions containing mutated primers and original primers were compared when the template concentration is 10^7^ copies/ml.

### Effect of Mismatch Between Probes and Template on RT-qPCR Reaction

In TaqMan RT-qPCR reaction, fluorescence is produced when Taq DNA Polymerase cuts off the fluorescent group-modified nucleotide at the 5’ end of the probe. Mutations were designed on the template DNA to make mismatches between the first nucleotide at the 5’ end and in the middle of the probe, respectively. At each position, the nucleotide was mutated into another three mismatched nucleotides (for example, A was mutated to T, C, and G). Then, the Ct values for the RT-qPCR reactions containing the mismatched DNA template and probe and the reaction containing the original DNA template and probe were compared, with the template concentration at 10^7^ copies/ml.

### Analysis of the Interactions Between a Specific Amino Acid Residue and Surroundings in N Protein

The interactions between a specific amino acid site (W108, W132, W301, M322) and surroundings was analyzed to evaluate their role in stabilizing protein structure. The molecular graphics figures were generated using PyMOL (http://www.pymol.org).

### Evaluation of the Sensitivity of Structure-Based Primers

To evaluate the sensitivity of the primers designed based on structure (NAm1 and NAm2), Ct values for the PCR reactions containing our primers and the *N* gene targeting primers designed by US and Chinese CDCs at four RNA template concentrations (10^7^, 10^6^, 10^5^, and 10^4^ copies/ml) were compared. All the RT-qPCR reactions contain a pair of primers at 400 nM and a probe at 200 nM. The RT-qPCR assays were performed on Analytik jena qTOWER3.

### Evaluation of the Specificity of Structure-Based Primers

To date, a total of seven types of coronaviruses have been identified as human pathogens (Zhu et al., 2020). Well-designed primers should be able to distinguish between different coronaviruses, especially between the highly pathogenic and the less pathogenic viruses. For specificity evaluation, the plasmids containing the *N* genes of the other six types of coronaviruses and human genome were prepared and used as templates at 10^7^ copies/ml in RT-qPCR reactions. The Ct values for RT-qPCR reactions containing different combinations of primers and templates were measured and compared. The data are obtained from triplicate experiments.

### Determination of the Limit of Detection

Under the same reaction system (different primer pairs), RT-qPCR was performed with five template concentrations of 10^3^, 700, 500, 400, and 300 copies/ml, respectively, and each concentration was repeated 20 times.

### Result Judgment

When Ct ≤38, it is judged as a positive sample; Ct >38 or no Ct is judged as a negative sample. The template concentration with a detection rate greater than 95% (19 times) was defined as the limit of detection of the primer pair.

### Protein Expression and Purification

For the protein expression of N-terminal domain (NTD), C-terminal domain (CTD), and their mutants, *E. coli* BL21(DE3) cells containing expression plasmids were grown in LB medium supplemented with 50 μg/ml kanamycin at 37°C, 200 rpm. When the OD600 reached 0.8, the temperature was lowered to 16°C, and then a final concentration of 0.5 mM isopropyl β-D-thiogalactopyranoside (IPTG) was added for overnight induction. For the expression of the N protein and its mutants, when the OD600 reached 0.8, IPTG at a final concentration of 0.5 mM was added to induce expression for 3 h.

For protein purification, cells were harvested by centrifugation at 5,000 *g* for 20 min. Cell pellet was resuspended in lysis buffer (25 mM Tris–HCl, pH 8.0, 500 mM NaCl) and then lysed by sonication on ice. After centrifugation at 28,370 *g* for 50 min at 4°C, the supernatant was loaded onto a ““nickel chelating sepharose affinity column (GE Healthcare) equilibrated with lysis buffer in advance. The column was then washed with wash buffer (25 mM Tris–HCl, pH 8.0, 500 mM NaCl, 50 mM imidazole) and then eluted with elution buffer (25 mM Tris–HCl, pH 8.0, 500 mM NaCl, 250 mM imidazole). The eluted sample was further purified by size-exclusion chromatography using Superdex 200 (GE Healthcare) in 25 mM Tris–HCl, pH 8.0, and 500 mM NaCl. Finally, SDS-PAGE was used to assess protein purity.

### Determination of Protein Thermal Stability

The effects of mutations on specific amino acid residues were assessed by measuring the thermal stability of the wild-type and mutant NTDs and CTDs. For this purpose, the measurement was performed by using Protein Thermal Shift™ Kit on Applied Biosystems QuantStudio 3. Then, 20 μl of protein sample at 0.4 µg/µl was heated from 25 to 99°C at a rate of 0.15°C/s. Data collected from triplicate experiments was used to calculate *T*_m_ values using Protein Thermal Shift™ Software 1.4 by fitting the data in the region of analysis to the Boltzmann equation.

### Statistics Analysis

All data were collected in at least three independent experiments and were presented as mean ± standard deviation of the results of triplicate experiments. The Student’s *t*-test was used to perform the statistical analyses. Statistical significance was assessed based on the *p*-value: *p < 0.05, **p < 0.01, and ***p < 0.001.

## Results

### The Mismatches That Occur at the 3′ End of the Primer Have the Greatest Impact on PCR Reaction

**Figure 1:**
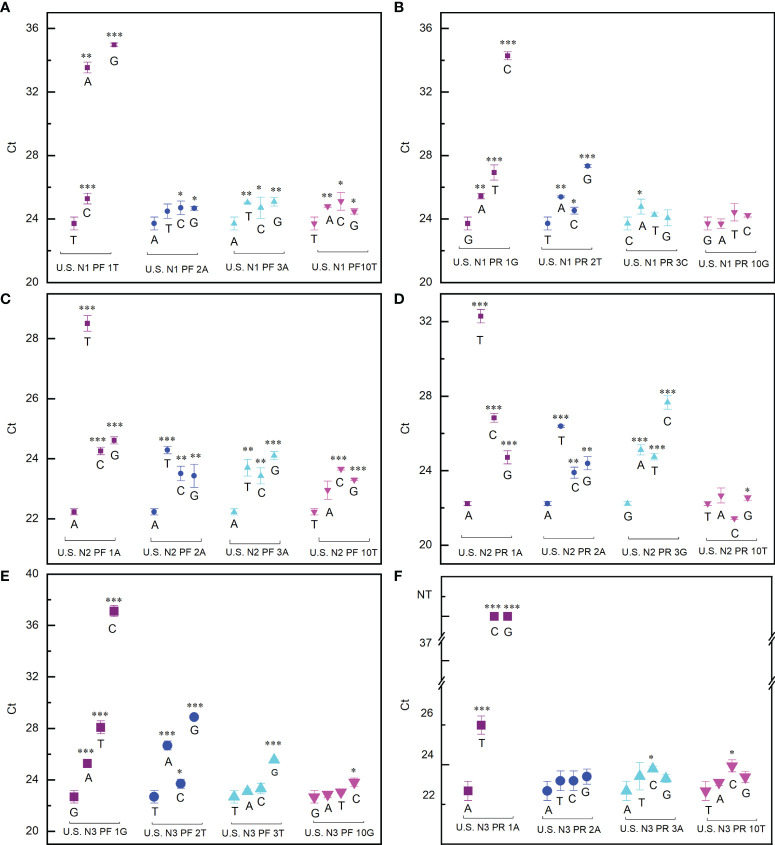
Effect of mismatch between primers and template on PCR reaction. **(A, B)** Ct values detected when the US N1 forward primer **(A)** and reverse primer **(B)** contained mismatch with the template. **(C, D)** Ct values detected when the US N2 forward primer **(C)** and reverse primer **(D)** contained mismatch with the template. **(E, F)** Ct values detected when the US N3 forward primer **(E)** and reverse primer **(F)** contained mismatch with the template. The purple square, blue dot, cyan upper triangle, and magenta lower triangle represent the first, second, third, and 10th (middle position) base of the 3′ end of the primer, respectively. Student’s *t*-test by SPSS 15 was used for data analysis. A *p*-value < 0.05 was considered statistically significant. *p < 0.05; **p < 0.01; ***p < 0.001.

To find the best strategy for designing primers which make a PCR reaction minimally affected by mutations, the effect of mutations at different positions has been extensively evaluated. The impact of the mutations is indicated by the difference of the cycle threshold (Ct) value with or without mismatch. Our data showed that, for all the six primers tested, when the mismatch occurred at the 3′ end the Ct value difference (ΔCt) reached the maximum (**Figure 1**). In the worst cases, for example, when the first nucleotide A at the 3′ end of the N3 forward primer was replaced by C or G, the PCR reaction completely failed (**Figure 1F**). This means that if the virus had a mutation at this position, the RT-qPCR test would always give a false-negative result even if the viral load in the sample is high. The ΔCt decreased as the mismatch moves away from the 3′ end of the primers (**Figure 1**). When the mismatch occurred in the middle of the primer, ΔCt is within ±1, indicating that the PCR test is basically unaffected.

**Figure 2:**
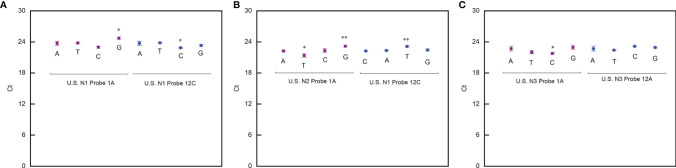
Effect of mismatches between probes and template on PCR reaction. **(A)** Ct values detected when the US N1 probe contained mismatch with the template. **(B)** Ct values detected when the US N2 probe contained mismatch with the template. **(C)** Ct values detected when the US N3 probe contained mismatch with the template. Student’s *t*-test by SPSS 15 was used for data analysis. A *p*-value < 0.05 was considered statistically significant. *p < 0.05; **p < 0.01.

By using a similar procedure, we also evaluated the impact of the mismatch occurring at different positions on the probes. The exciting thing is that the PCR reaction is not sensitive to the mismatch on the probes (ΔCt is within ±1) (**Figure 2**). This means that a strategy to design primers for a robust PCR reaction not sensitive to virus mutation is to place the 3′ end of the primers at the positions where any mutation is fatal to the virus. In this way, the mutations that significantly affect the RT-qPCR test will never happen.

In order to achieve this goal, the primers should be designed based on both the nucleic acid sequence and the structure of the protein encoded. The three nucleotides closest to the 3′ end of the primer should be the codon which encodes tryptophan in the structure core. Tryptophan is encoded by only one codon, so any mutation at that location alters the amino acid residue. Since tryptophan plays an important role in stabilizing protein structure (Ohmae et al., 2001), the mutation of tryptophan will lead to the destruction of protein structure stability. This means that this kind of SARS-CoV-2 variant could not survive.

### Primers Targeting the *N* Gene Were Designed Based on Both Gene Sequence and Protein Structure

**Figure 3:**
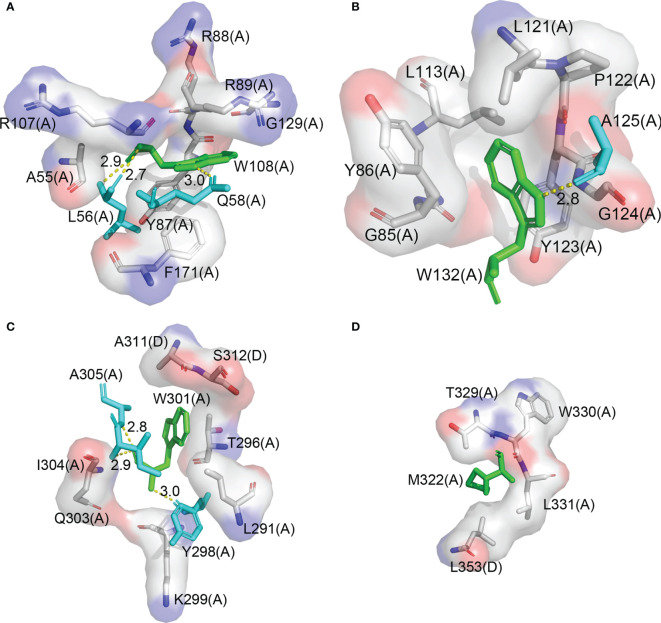
W108, W132, W301, and M322 are in the structure core of the N protein. **(A)** The interaction diagram of W108 in the N protein structure. **(B)** The interaction diagram of W132 in the N protein structure. **(C)** The interaction diagram of W301 in the N protein structure. **(D)** The interaction diagram of M322 in the N protein structure.

*N* gene is the major target of the RT-qPCR test for COVID-19 diagnosis. It encodes the N protein, which consists of a NTD and a CTD, and forms a dimer in solution through the dimerization of CTD (Peng et al., 2020). NTD contains two tryptophan residues, W108 and W132, which are located right in the structure core. W108 forms a hydrogen bond with Q58 and L56 and forms a hydrophobic interaction with A55, Y87, R88, R89, R107, G129, and F171, respectively (**Figure 3A**). W132 forms a hydrogen bond with A125 and forms a hydrophobic interaction with G85, G124, Y123, Y86, L113, L121, and P122, respectively (**Figure 3B**). A pair of primers were designed, with their 3′ end located on the codon of the two tryptophans, respectively. CTD contains only one tryptophan residue W301. Fortunately, there is M322 which is at the right distance from W301. Methionine also has only one codon, so we choose it as the suboptimal choice and design the second pair of primers. Both W301 and M322 are located on the dimer interface. W301 forms a hydrogen bond with Y298, I304, and A305 and forms a hydrophobic interaction with K299, Q303, Y296, and I291 in the same protomer and with A311 and S312 in the other protomer, respectively (**Figure 3C**). M322 forms a hydrophobic interaction with W330, L331, T329, and l353 in the same protomer and with L353 in the other protomer, respectively (**Figure 3D**). Based on the structural information of the *N* protein, we selected W108, W132, W301, and M322 for primer design because these residue pairs are not only critical for the stability of the protein but also of the right distance on the sequence for a quick PCR reaction. We expect that any mutation that changes one of these amino acid residues would result in an inactive N protein.

### Effect of Mutations on the Thermal Stability of N Protein

**Figure 4:**
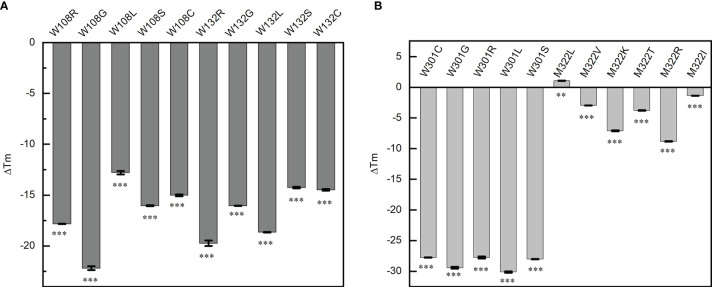
Point mutations affect the thermal stability of the corresponding domains. **(A)** Thermal transition midpoint temperature difference of the mutant domains at positions W108 and W132. **(B)** Thermal transition midpoint temperature difference of the mutant domains at positions W301 and M322. Student’s *t*-test was used to conduct statistical analysis, and differences were considered significant when *p <*0.05, **p < 0.01, and ***p < 0.001.

Single-base mutations are much more likely to occur than double- or triple-base mutations during virus replication, for example, the famous P4715L in *ORF1ab* (nucleotide 14,143, C to T) and D614G in *S* (nucleotide 23,403, A to G) all resulted from a single-base mutation. The probability of double- or triple-base mutations on the codon of the same amino acid is very small, so we tried to investigate if all the single-nucleotide mutations in the codon of W108, W132, W301, and M322 would produce an inactive N protein. These mutations totally produce 21 mutants of the N protein domain (except for the stop codon). The thermal transition midpoint temperature (*T*_m_) of mutant NTDs and CTDs were measured and compared with the corresponding wild-type domains. Compared with the wild-type NTD, the *T*_m_ values of W108C, W108S, W108L, W108R, W108G, W132C, W132S, W132L, W132R, and W132G mutant domains decreased by 15.02 ± 0.09, 16.04 ± 0.06, 12.8 ± 0.18, 17.82 ± 0.02, 22.19 ± 0.19, 14.48 ± 0.08, 14.25 ± 0.08, 18.65 ± 0.02, 19.74 ± 0.28, and 16.05 ± 0.02°C, respectively (**Figure 4A**). Compared with the wild-type CTD, the *T*_m_ values of W301C, W301S, W301L, W301R, W301G, M322V, M322K, M322R, M322T, and M322I mutant domains decreased by 27.76 ± 0.04, 28.02 ± 0.05, 30.13 ± 0.11, 27.74 ± 0.17, 29.42 ± 0.16, 2.955 ± 0.01, 7.095 ± 0.10, 8.82 ± 0.05, 3.79 ± 0.05, and 1.38 ± 0.01°C, respectively. Unexpectedly, the *T*_m_ value of M322L mutant domain increased by 1.085°C (**Figure 4B**).

All the mutations at W108, W132, and W301 and most mutations at M322 caused a significant decrease of the thermal stability of the NTD or CTD. It was indicated that all these three tryptophan residues are critical for the thermal stability of N protein. Any mutation occurring on any of these tryptophan would decrease the *T*_m_ value to below 37°C, which means that these mutants would have no normal functions inside the human body.

### The N Protein Mutants Are Poorly Expressed at 37°C

**Figure 5:**
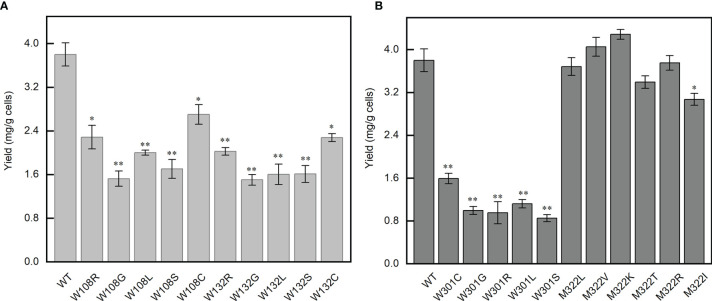
The yield of N protein mutants expressed at 37°C. **(A)** The yield of the mutants at positions 108 and 132. **(B)** The yield of the mutants at positions 301 and 322. Statistical significance is indicated as compared with wild type using *t*-test. *p < 0.05; **p < 0.01.

Since the mutations on W108, W132, and W301 cause the *T*_m_ values of the corresponding domain to be lower than 37°C, we tested if the full-length protein containing these mutations could be expressed normally. The effect of these mutations on the N protein was evaluated by comparing their expression and solubility with the wild-type protein. The results showed that all mutations on W108, W132, and W301 caused a significant decrease in protein expression level and solubility. The mutant W301 has the lowest solubility (**Figures 5A, B**). By contrast, the mutations on M322 do not affect the protein expression level (**Figure 5B**). In addition, all these mutant proteins have a poorer behavior in comparison with the wild-type protein. These results, combined with the data of thermal stability of the mutant domains, indicate that the sites that we selected are critical to the function of the N protein. Therefore, SARS-CoV-2 variants containing these kinds of mutations would be outcompeted by other strains inside the human body and have no chance to influence the RT-qPCR test.

### Performance of the Structure-Based Primers

We have shown that if a PCR reaction contains structure-based primers it will be very difficult for a virus to evade the RT-qPCR test by mutation. Now we need to know if the primers designed by this strategy perform as well as the primers designed by both US and Chinese CDCs. Based on the structure of the N protein, we designed two pairs of primers, N Anti-mutation 1 (NAm1) and N Anti-mutation 2 (NAm2), targeting NTD and CTD, respectively. The 3′ end of the forward primer of NAm1 is located on the nucleotide sequence encoding W108, and the 3′ end of the reverse primer is located on the nucleotide sequence encoding W132. For NAm2, the 3′ end of the forward and reverse primers are on the nucleotide sequences encoding W301and M322, respectively. The sequences and *T*_m_ values of the primers and probes are shown in **Table 1**.

**Table 1:** The sequences of N anti-mutation (NAm) primers and probes.

Primers	Sequence	*T*_m_ (°C)
NAm1 PF	5′-ATGAAAGATCTCAGTCCAAGATGG-3′	57
NAm1 PR	5′-CTCCCTCAGTTGCAACCCA-3′	58
NAm1 Probe	5′-FAM-CTTCCCTATGGTGCTAACAAAGACGGC-BHQ1 -3′	62
NAm2 PF	5′- CAAGGAACTGATTACAAACATTGG -3′	54
NAm2 PR	5′-CCGAAGGTGTGACTTCCAT-3′	56
NAm2 Probe	5′-FAM-CAGCGCTTCAGCGTTCTTCGGA-BHQ1 -3′	63

**Figure 6:**
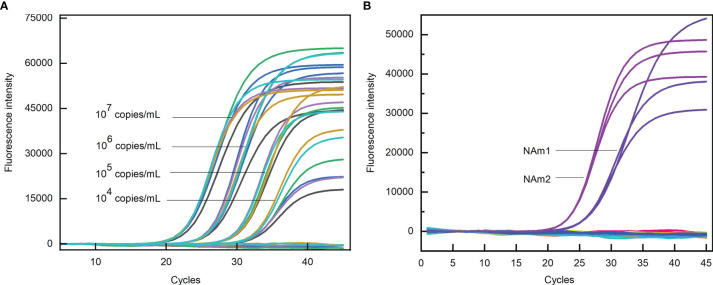
**(A)** Sensitivity test of N anti-mutation (NAm) primers. The black, green, blue, cyan, orange, and purple curves represent US N1, N2, N3, CHN N, NAm1, and NAm2 primers, respectively. **(B)** Specificity test of NAm primers. The blue and purple curves represent the amplification curves of NAm1 and NAm2 when the SARS *N* gene was used as the template, and the flat lines represent the amplification curves when the MERS-CoV, HCoV-229E, HCoV-OC43, HCoV-NL63, and HCoV-HKU1 *N* gene and human genome were used as templates.

The sensitivity of the primers and probes were evaluated by PCR reaction containing a certain amount of the template. The Ct values obtained for NAm1 and NAm2 were compared with the average Ct values obtained for four pairs of primers US N1, US N2, US N3, and CHN N. The results showed that the differences of the Ct values were all within ±1 at four template concentrations (**Figure 6A**). Both NAm1 and NAm2 meet the limit of detection (500 copies/ml) of the primers designed by the US and Chinese CDCs (**Table 2**). This means that NAm1 and NAm2 have the same sensitivity as the primers designed by the US and Chinese CDCs.

**Table 2:** Positive rate for primer pairs at different concentrations.

Primer pairs	Sample concentration (copies/ml)				
	1,000	700	500	400	300
US N1	100%	100%	95%	35%	15%
US N2	100%	100%	95%	45%	15%
US N3	100%	100%	100%	55%	20%
CHN N	100%	100%	95%	40%	10%
NAm1	100%	100%	100%	55%	20%
NAm2	100%	100%	95%	50%	15%

The specificity of the primers and probes were evaluated by PCR reaction containing a certain amount of the *N* gene of SARS-CoV-2, SARS-CoV, MERS-CoV, HcoV-229E, HCoV-OC43, HCoV-NL63, and HCoV-HKU1 and human genome. In the PCR assay, when the concentration of the SARS-CoV *N* gene template was 10^7^ copies/ml, the Ct values for NAm1 and NAm2 were 24.82 and 22.78, respectively. However, when the same amount of MERS-CoV, HCoV-229E, HCoV-OC43, HCoV-NL63, and HCoV-HKU1 *N* genes and human genome was used as template, no amplification occurs (**Figure 6B**). Since the SARS virus has not been identified in the world since 2003, we thought that the specificity of NAm1 and NAm2 is both excellent.

## Discussion

The pandemic of COVID-19 is still raging in many countries. It not only makes great damages to many developed countries but also makes catastrophe in developing countries like India and other countries in south Asia (Li et al., 2020). However, except for intravenous remdesivir and dexamethasone, which have modest effects in moderate to severe COVID-19, no strong clinical evidence supports the efficacy of any other drugs against SARS-CoV-2 (Asselah et al., 2021). Vaccines are the most effective strategy to prevent infectious diseases, but the limited production means it may take years to vaccinate the majority of the population on earth. Therefore, before effective treatment and vaccines can contain the pandemic successfully, detection and isolation of people infected with SARS-CoV-2 are the first and most effective steps to curb the spread of SARS-CoV-2.

**Figure 7:**
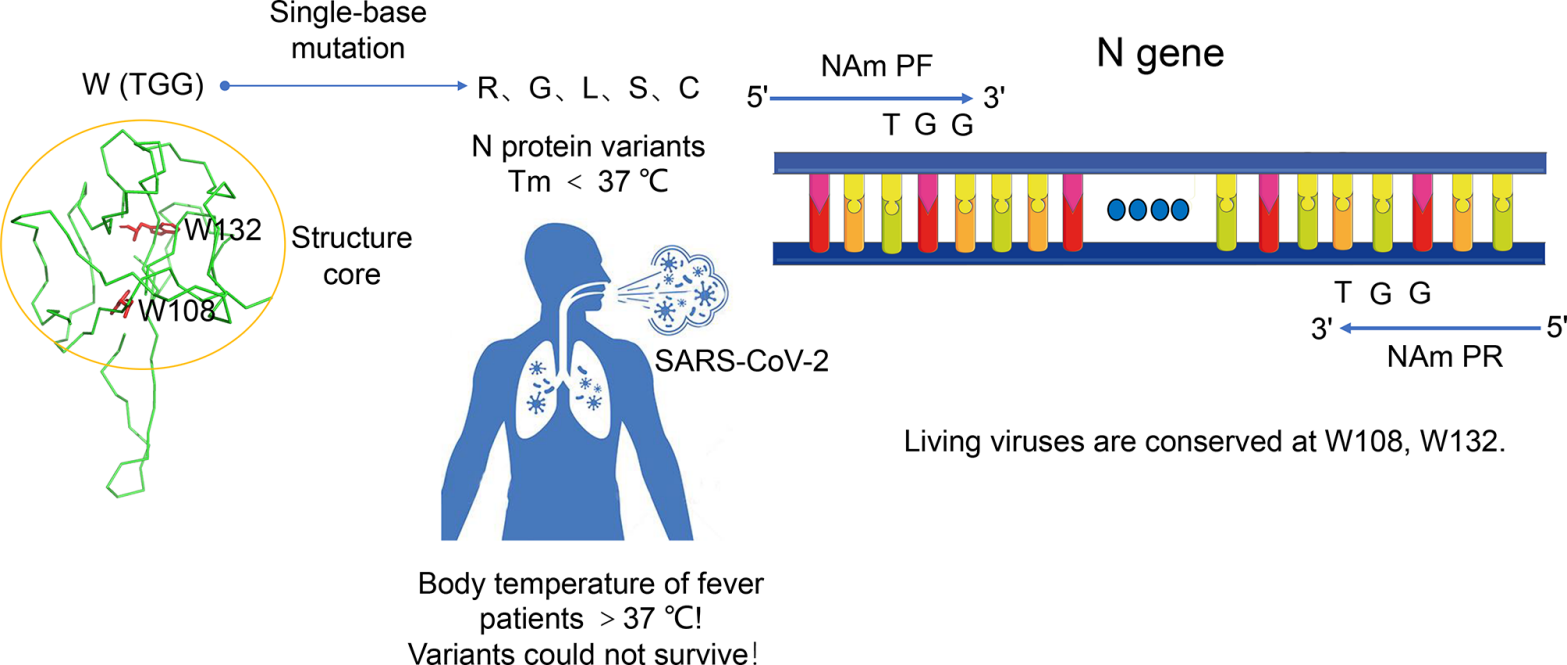
Primer design principle based on protein structure and amino acid codon.

By far, RT-qPCR is the golden standard for COVID-19 test. The success of RT-qPCR depends first and foremost on the performance of the primers and further on the sequence matching between the primers and SARS-CoV-2 genome. The primers currently used are all based on the earliest version of SARS-CoV-2 genome sequence. However, SARS-CoV-2 constantly undergoes mutations. The mutations in S protein may change the antigenicity, leading to immune escape and affecting vaccine design (Naqvi et al., 2020; Silveira et al., 2021). There is no doubt that, once mutations occur in the RT-qPCR-targeted region, the RT-qPCR test may give a false-negative result^19^. Since thousands of single-nucleotide variants have been identified by far and the number of mutations is still growing rapidly, the possibility for the appearance of some variants that can evade the RT-qPCR test is also becoming increasingly higher. To avoid this situation, we suggest an innovative primer design strategy such that the primers should be designed based on both the nucleic acid sequence and the structure of the protein encoded (**Figure 7**). By using this strategy, we designed two pairs of primers targeting the *N* gene. Experiments showed that these primers have the same sensitivity and high specificity as the primers designed by US and Chinese CDCs. More importantly, any mutation affecting the performance of these primers (especially NAm1) would lead to the production of a protein losing structural stability and thus an inactive virus variant. *N* protein contains five tryptophans (W52, W108, W132, W301, and W330) and seven methionines (M1, M101, M210, M234, M317, M322, and M411). By analyzing the three-dimensional structure of *N* protein, we can easily figure out which residues are in the structure core and choose them for primer design. By this means, we need not try every Trp and Met to find the best place, thus greatly reducing the workload. This strategy would be much more convenient when the target protein is much bigger. Therefore, the primer pairs that we designed were the product of integrating the gene sequence and the three-dimensional structure of the protein rather than simply based on the gene sequence and degeneracy of codons.

Since SARS-CoV-2 has many genes, it is not difficult to design primers to target other genes based on the same principle and form a multi-channel PCR system. This way, the risk of PCR failure caused by SARS-CoV-2 mutations is minimized. Furthermore, we believe that this strategy can also be used to design primers for the diagnosis of other viruses and even bacterial or fungal pathogens. Since all pathogens have numerous variants and only a small fraction of them are sequenced, the new strategy for primer design will no doubt ease the concerns that the RT-qPCR test may fail to detect certain variants.

## Data Availability Statement

The raw data supporting the conclusions of this article will be made available by the authors, without undue reservation.

## Author Contributions

LG conceived and directed this research, is the guarantor of this work, has full access to all of the data in the study, and takes responsibility for the integrity of the data and the accuracy of the data analysis. HD designed the methods. HD, SW, and JZ performed most of the experiments and wrote the manuscript. KZ, FZ, and HW helped with the experiments. SX and WH helped with the project and the writing. All authors contributed to the article and approved the submitted version.

## Funding

This research was supported by the Shandong Provincial Key Research and Development Program (2020CXGC011305).

## Conflict of Interest

JZ and SX were employed by Shandong Shtars Medical Technology Co., Ltd.

The remaining authors declare that the research was conducted in the absence of any commercial or financial relationships that could be construed as a potential conflict of interest.

## Publisher’s Note

All claims expressed in this article are solely those of the authors and do not necessarily represent those of their affiliated organizations, or those of the publisher, the editors and the reviewers. Any product that may be evaluated in this article, or claim that may be made by its manufacturer, is not guaranteed or endorsed by the publisher.
